# Weaving Vectorial Responses: Magnetorheological Fibrous Materials for Programmable Sensing and Actuation

**DOI:** 10.3390/s26030865

**Published:** 2026-01-28

**Authors:** Yunfei Tang, Jianmin Li

**Affiliations:** 1 College of Electronic and Optical Engineering and College of Flexible Electronics (Future Technology), Nanjing University of Posts and Telecommunications, 9 Wenyuan Road, Nanjing 210023, China; 1025020439@njupt.edu.cn; 2 State Key Laboratory of Flexible Electronics (LoFE) and Institute of Advanced Materials (IAM), Nanjing University of Posts and Telecommunications, 9 Wenyuan Road, Nanjing 210023, China

**Keywords:** magnetorheological fibrous materials, vector-stimuli-responsive, soft magnetic materials

## Abstract

Magnetorheological (MR) materials, with the ability of vectorial response, offer exciting opportunities for next-generation wearables and soft robotic systems. Although some existing MR materials and fiber designs can produce directional responses, they typically rely on strategies—such as hard-magnetic loading or pre-magnetization—that constrain safety and large-scale manufacturability. This Communication highlights a paradigm-shifting advance reported by Pu et al., that a soft-magnetic fibrous architecture achieves genuine vector-stimuli-responsiveness under low, safe magnetic fields without pre-magnetization. We articulate the great breakthrough of this work through a hierarchical design framework, demonstrating how the synergistic innovation at the material (magnetic dipole aligned in low-density polyethylene), fiber (drawing-induced magnetic easy axis), yarn (twist-induced cooperative effects), and fabric (vertical or horizontal magnetic field response capability) levels collectively resolves the longstanding trade-offs between performance, manufacturability, and safety. As a result, this strategy demonstrates strong universality in terms of materials, although only the carbonyl iron particles were used. This approach not only enables programmable bending, stiffening, shear, and compression in textiles but also establishes a versatile platform for magneto-programmable systems. Furthermore, we delineate the critical challenges and future trajectories—from theoretical modeling and integration of complementary stimuli to the development of three-dimensional textile architectures—that this new platform opens for the fields of haptics, soft robotics, and adaptive wearables.

## 1. Introduction

Stimuli-responsive fibers and textiles are a class of functional materials for intelligent manufacturing, wearable technologies, and soft robotics, owing to their flexibility and lightweight nature, as well as the ability to convert external energy inputs into mechanical responses. In most existing systems, the stimulus–response relationship is fundamentally scalar: although the applied stimulus may possess a vector nature, the material response depends solely on its magnitude and can be represented by a single scalar variable. Over the past decades, researchers have developed a wide range of stimulus-responsive fiber materials—based on shape-memory polymers, dielectric elastomers, and other materials—that react to temperature, humidity, light, or electric fields, enabling tunable electrical, mechanical, and optical properties [1,2,3,4,5,6,7]. While these scalar-responsive materials exhibit tunable properties, these systems can only sense or quantify the stimulus intensity and therefore lack directional controllability and functional diversity [4,8]. As a result, such systems are unable to achieve the complex, vector-defined mechanical actions such as direction-selective bending, shearing, or stiffness modulation.

Beyond purely scalar responses, a class of stimulus-responsive materials exhibits direction-dependent mechanical behavior, in which the material response varies with the orientation of the applied stimulus relative to an internal structural anisotropy. Magnetorheological (MR) materials, composed of magnetic particles dispersed in a viscoelastic matrix, convert magnetic stimuli into tunable mechanical responses [9,10]. When exposed to a magnetic field, the magnetized particles attract each other through dipole–dipole interaction to form fiber-like structures, known as the MR effect, that increase the viscosity and stiffness of the MR materials [11]. One approach relies on anisotropic MR elastomers, in which predefined soft-magnetic microstructures are embedded within the polymer matrix. These systems exhibit direction-dependent responses to magnetic fields [12,13,14]. However, these materials are usually limited by their inherent rigidity, which requires a high magnetic field (>500 mT, unsafe for the human body [15]) to trigger the response [16,17,18]. In addition, when integrated into textile architectures, the performance of the fabrics is constrained by the combined effects of inter-fiber magnetic interactions, the limited precision of textile manufacturing, and the intrinsically non-bonded, hierarchical nature of fabrics [19,20]. In parallel, magnetic fibers containing hard-magnetic particles have been explored to achieve direction-dependent actuation through direct magnetization. In this case, the pre-magnetization process is often limited by the magnetizer’s spatial constraints, which restrict the textile size to a few centimeters [21]. Moreover, magnetic interactions between magnetized parts during actuation hinder stable, controllable and reversible movements, which occur in fabrics that allow hierarchical internal relative motion.

**Figure 1:**
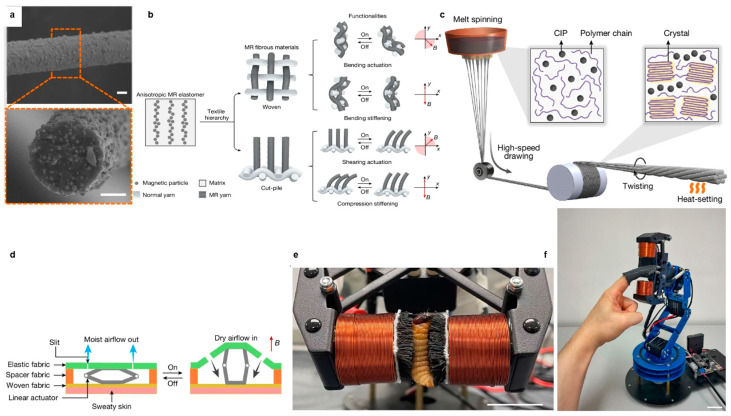
Design and fabrication of MR fibrous materials with vectorial mechanical actuation functionalities and demonstration of smart textiles based on MR fabrics: (**a**) Side and cross-sectional views of the MR fiber. Scale bars, 20 μm (**b**) Structure of fibrous MR materials composed of magnetic particles dispersed in an elastomeric matrix, enabling vector-responsive actuation. (**c**) Schematic of the production of intrinsic magnetic anisotropic MR fabrics, which can be used for mass production. (**d**) Structure and working principle of the active ventilation fabric driven by the linear actuator. (**e**) Close-up of the gripper holding a live worm (1 A input current). (**f**) An untethered haptic finger glove remotely controlled by the mobile magnetic actuation system for haptic feedback.

To move beyond direction-dependent actuation toward a true vectorial response, a stimulus-responsive material must enable controlled mechanical outputs such that both the magnitude and direction of the mechanical output can be independently and reversibly regulated by the orientation and strength of the applied stimulus. In this context, the work reported by Pu et al. introduce a class of anisotropic MR fibrous materials that realize true vectorial mechanical responses while retaining high flexibility and multimodal actuation. These soft-magnetic systems exhibit directionally controlled bending and field-tunable stiffness under relatively low magnetic fields (<300 mT), enabling programmable, reversible actuation. The material design is based on soft-magnetic MR fibers containing a dense population of carbonyl iron particles (CIPs) embedded within a compliant polymer (low-density polyethylene, LDPE) matrix (Figure 1a,c). During post-processing, drawing induces strong axial alignment of the CIPs, establishing a pronounced magnetic easy axis that governs the field-oriented mechanical response [22]. Importantly, adopting soft-magnetic particles allows the MR composite to be processed as continuous fibers—overcoming the size and geometry limitations of conventional MR materials that rely on pre-magnetization or rigid elastomeric matrices. As a result, the fibers can be produced in long lengths, twisted into cooperative MR yarns, and woven or tufted into large-area textiles without loss of magnetic responsiveness. This hierarchical integration from fiber to yarn to fabric provides a structural basis for amplifying and preserving vectorial magnetic actuation across multiple length scales (Figure 1b).

## 2. Design Principles of Vectorial MR Fibrous Systems

At the fiber level, the vector-stimuli responsiveness arises from the aligned magnetic dipoles embedded within the polymer matrix. Owing to the drawing-induced axial alignment, the dipoles share a common orientation along the fiber axis, endowing the fiber with a well-defined magnetic easy axis. During melt spinning and drawing, polymer chains are highly oriented and partially converted into extended-chain crystals (Figure 1a), resulting in enhanced stiffness and thermal stability. When an external magnetic field is applied at an angle relative to this axis, the resulting angular misalignment produces a magnetic torque that competes with the viscoelastic resistance of the polymer matrix, leading to controlled fiber bending toward field alignment. Importantly, the mechanical response of the fiber varies continuously with the orientation of the applied field, rather than switching between discrete modes. This angle-dependent coupling between magnetic torque and viscoelastic deformation enables each individual fiber to transduce the direction of the magnetic stimulus into a deterministic mechanical output. As such, these fibers constitute the fundamental building blocks of vector-controlled actuation.

At the yarn level, MR yarns were fabricated by twisting seven MR fibers and subsequently heat-setting the assembly to stabilize the helical structure (Figure 1c). Twisting releases axial stress and promotes chain relaxation and re-folding into oriented folded-chain lamellae (Figure 1a), which stabilizes the microstructure while preserving molecular orientation. This hierarchical structural evolution, combined with the yarn-level architecture, yields a composite fiber with balanced stiffness, strength, ductility, and superior fatigue resistance. When multiple fibers are twisted into a helical yarn, the mechanics no longer correspond to a simple sum of individual fiber responses. Instead, cooperative magnetic dipole interactions and helical geometric coupling produce emergent actuation modes that exceed the capabilities of a single fiber. The first is the bending mode: the collective misalignment of the fiber easy axes generates an amplified magnetic torque under an external magnetic field applied at an oblique angle to the yarn axis, leading to pronounced yarn-level bending. The second mode is the stiffening mode: when a field is aligned parallel to the yarn axis, magnetic attraction between the helically wrapped fibers constrains relative sliding and rotation, leading to an increase in bending rigidity. Importantly, the transition between these deformation modes is governed continuously by the orientation of the applied field, rather than by discrete switching. As a result of this helical geometry-enabled magnetic coupling, MR yarns function as lightweight soft actuators capable of lifting loads orders of magnitude greater than their own mass, while remaining flexible enough to be tied, knotted, and processed using standard textile methods.

At the fabric level, two distinct textile architectures were employed—woven and cut-pile structures—to organize and preserve vectorial magnetic actuation at the macroscopic responses (Figure 1b). By translating yarn-scale anisotropic and vector-responsive behavior into spatially structured textile geometries, these architectures enable controlled, application-specific vertical or horizontal vectorial mechanical outputs. Woven fabrics, created by interlacing MR yarns at right angles, form planar, grid-like networks. This classic textile geometry excels in harnessing in-plane, horizontal vectorial responses. The interlaced structure allows the fabric to act as a cohesive sheet that undergoes controlled bending, folding, or linear contraction when subjected to transverse magnetic fields, making it ideal for applications like dynamic shape-changing surfaces and linear fabric actuators. Conversely, cut-pile fabrics adopt a radically different approach by embedding MR yarns vertically into a backing substrate, akin to a field of magnetic bristles. This architecture fundamentally reorients the primary actuation axis to the out-of-plane, vertical direction. It enables two unique modes: magnetically tunable compression stiffness when a field is applied perpendicular to the fabric plane, and in-plane shear displacement when a field is applied at an angle. This makes cut-pile structures uniquely suited for adaptive cushioning, conformal gripping, and haptic interfaces requiring localized pressure modulation. Thus, the choice of textile architecture becomes a high-level design parameter, deterministically mapping magnetic field vectors into desired macroscopic deformation fields. Calculations show that the magnetic moment density of a single straight yarn reaches its maximum when the yarn axis is oriented at 45° relative to the external magnetic field, where the vector components of magnetization and mechanical torque are optimally coupled. However, interlacing the yarns into woven structures slightly reduces the moment density, primarily due to the crimped geometry that introduces local misalignments from the ideal 45° orientation [22]. Nevertheless, woven fabrics inherently adopt right-angle interlacing, which—while not magnetically optimal—provides mechanical integrity, uniform stress distribution, and scalability, while still preserving sufficient angular diversity for vectorial responses. Through this architecture, microscopic dipole anisotropy is geometrically organized into macroscopic vectorial deformation fields—allowing woven fabrics to exhibit vector-controlled bending and contraction under transverse fields, and cut-pile fabrics to realize programmable vectorial shear and compression—while retaining the softness, drape, and wearability.

## 3. Experimental Validation of Vectorial Responses

The vectorial actuation capability of the MR fibrous system is further substantiated through quantitative mechanical characterization across multiple deformation modes. Under a modest magnetic field of 280 mT, the bending stiffness of yarns increases by nearly 30-fold (from 0.68 to 20 mN·mm^2^), while achieving a bending moment density of 6.5 N·m·kg^−1^, sufficient to lift an object 185 times its own weight. Extending this yarn-level performance to the textile scale, the constructed woven and cut-pile MR fabrics exhibit distinct, programmable macroscopic responses governed by the applied magnetic field. The woven fabric exhibits reversible bending and linear contraction with a fast response time of 0.07–0.22 s and full recovery within 0.6 s, while the cut-pile configuration supports magnetically tunable shear and compression, with the compressive modulus rising from 1 to 22.5 kPa under 280 mT and generating shear forces up to 110 mN. Both fabrics maintain their softness and mechanical performance after more than 10,000 cycles, underscoring their exceptional durability and fatigue resistance required for practical vector-controlled systems. Taken together, these results show that changing the orientation of the applied magnetic field systematically selects distinct deformation modes—bending, contraction, shear, or compression—within the same material system, confirming a genuine vectorial mechanical response rather than isolated direction-dependent effects.

These programmable vectorial responses enable a range of proof-of-concept demonstrations that exploit different deformation modes within the same material platform. Importantly, all these vectorial responses occur well within safe magnetic field strengths for wearable applications, enabling direct translation into functional prototypes. A magnetically actuated textile enabled active ventilation by dynamically tuning its porosity, thereby regulating airflow and moisture permeability in real time (Figure 1d). A soft gripper constructed from MR yarns could gently grasp fragile objects such as tofu and blueberries without damage, highlighting precise, field-controlled deformation (Figure 1e). Moreover, a lightweight, remotely controlled haptic glove integrating both cut-pile and plain-weave MR fabrics delivered programmable kinesthetic and tactile feedback by switching between soft and rigid states through direct magnetic actuation (Figure 1f). Operating via a mobile magnetic system that modulates the relative position of electromagnets and MR fabrics, the glove provides tunable stiffness and contact pressure without the need for bulky motors or pneumatic components, resulting in a comfortable and natural wearing experience.

## 4. Discussion

Beyond the specific material combination employed, the proposed vectorial actuation mechanism establishes a generalizable framework for magnetic fibrous materials. This framework relies on three conceptually distinct yet functionally coupled elements: (i) soft-magnetic fillers with high reversible susceptibility, (ii) a compliant polymer matrix that permits viscoelastic deformation under magnetic torque, and (iii) a processing-induced structural anisotropy that defines a stable magnetic easy axis. While the CIP/LDPE system provides a favorable balance of magnetic response, processability, and mechanical compliance, the underlying principle is not intrinsically limited to this pairing. Alternative soft-magnetic fillers, such as iron nanoparticles or magnetic nanorods, may offer different dipolar interaction strengths, dynamic response, and alignment stability [23], while different polymer matrices (e.g., elastomeric, thermoplastic) with tunable viscoelasticity could further modulate response speed, reversibility, and energy dissipation. Importantly, the present work demonstrates that vectorial actuation emerges primarily from the controlled coupling between magnetic anisotropy and mechanical compliance, rather than from material-specific magnetic remanence or pre-magnetization. In this sense, the MR fibrous architecture reported by Pu et al. defines a transferable design strategy, in which material selection governs performance envelopes (force, speed, modulus), whereas the fundamental capability of vectorial response is dictated by the hierarchical, anisotropic textile structure itself.

While this study represents a major step toward realizing vectorial mechanical responses in MR materials, several fundamental and practical issues remain to be clarified. At the material and fundamental level, a comprehensive quantitative model coupling magnetic dipole alignment, polymer viscoelastic relaxation, and helical yarn geometry remains an open challenge. Specifically, the type of magnetic fillers and their morphology (e.g., spherical particles, rods, or wire-like structures) are expected to critically influence dipolar interaction strength, alignment stability, and effective magnetic torque generation, yet are not currently incorporated into a unified quantitative framework [24,25]. In addition, the viscoelastic relaxation of the LDPE matrix governs the mechanical energy dissipation and the timescale for the reorientation of embedded CIPs following a change in the magnetic field vector. This time-dependent mechanical behavior directly influences the stability and reversibility of dipole alignment, which is the core of vectorial actuation. Concurrently, the helical geometry of the yarn intricately modulates this interaction: it dictates the spatial distribution of magnetic attraction between fibers (affording the observed stiffening via friction), transforms individual fiber bending torques into a collective yarn torque, and imposes geometric constraints on dipole rotation. The coupling between these domains is inherently multi-scale and nonlinear. For instance, magnetic interactions can locally alter polymer chain mobility, while stress fields induced by twisting can affect the effective magnetic anisotropy. Developing a coupled magneto-viscoelastic-structural model is therefore crucial to quantitatively predict performance metrics—such as the precise bending angle as a function of field direction, the hysteretic loss during cyclic actuation, or the frequency-dependent stiffening ratio—and to enable the inverse design of yarns with customized actuation signatures.

At the practical level, there are several possible pathways that could further expand the capabilities of MR textile systems. One direction is the integration of miniaturized, flexible permanent magnets or hybrid magnetic components, which would enable self-contained, untethered devices without dependence on external field sources. Another opportunity lies in coupling magnetic actuation with additional stimuli—thermal, electrical, optical, or chemical—to create multifunctional, reconfigurable soft systems capable of synergistic actuation modes and context-adaptive responses. Beyond functional diversification, advances in textile architecture offer another important frontier. Transitioning from planar woven fabrics to three-dimensional knitted, braided, or embroidered structures could unlock richer deformation pathways, enhanced load distribution, and bio-inspired actuation behaviors. In parallel, several practical considerations must be addressed for the deployment of the MR textiles in wearable or robotic settings. First, biocompatibility and long-term safety require thorough investigation. While the magnetic fields used are within safety guidelines, the implications of prolonged skin contact with the composite materials, potential allergenicity, and the biodurability under cyclic magnetic stimulation need a comprehensive study. Second, environmental stability and robustness are crucial for real-world operation. The performance of the MR fabrics under varying humidity, temperature extremes, and during mechanical abrasion—common in wearables and robotics—must be characterized and engineered for resilience. Third, challenges of system integration and ergonomics must be overcome. This includes the development of compact, lightweight, and flexible field sources (e.g., integrated permanent magnets or micro-coils), ensuring user comfort by managing the weight and stiffness of the active textile system, and maintaining consistent actuation performance during body movement or object manipulation. Finally, long-term durability and performance decay beyond 10,000 laboratory cycles need to be understood. Factors such as particle–polymer interface degradation, gradual fiber fatigue, and potential loss of magnetic dipole alignment over years of use are critical for determining product lifetime and reliability. Addressing these interdisciplinary challenges—spanning materials science, biomedical engineering, and design—is essential to transition this remarkable platform from compelling prototypes to trusted, functional technologies.

## 5. Conclusions

In summary, the work by Pu et al. signifies a pivotal shift in the landscape of stimuli-responsive materials, encoding the vectorial stimulus–response capabilities into the very fabric of textiles. This achievement transcends the development of a singular high-performance material, which establishes a hierarchical design framework where function emerges from the synergistic coupling of soft-magnetic anisotropy, viscoelastic matrix properties, and programmable textile architecture across fiber, yarn, and fabric scales. By eschewing pre-magnetization and leveraging low-field, soft-magnetic responses, it decouples sophisticated motion control from bulky external hardware. The demonstrations—from adaptive grippers to untethered haptic interfaces—are compelling validations of this principle, pointing toward a future where “smart fabrics” are truly dynamic, mechanical systems. Looking forward, this platform raises as many questions as it answers, charting clear directions for future inquiry. The immediate scientific frontier lies in developing coupled magneto-mechanical- viscoelastic models to predictively design performance. On the engineering side, the integration of miniaturized and flexible field sources, alongside the exploration of 3D textile architectures (knits, braids), will be crucial to unlock fully autonomous and complex shape-morphing systems. Furthermore, the material generality of the principle invites exploration of alternative filler–matrix combinations to tailor dynamic responses for specific applications, from medical exosuits to reconfigurable metamaterials. Ultimately, this work redefines the scope of functional textiles. It moves beyond static smartness or passive response, offering a blueprint for creating soft, distributed machines whose motions can be programmed by the structure of a field.

## Data Availability

Data are contained within the article.

## Abbreviations

The following abbreviations are used in this manuscript:

MR	Magnetorheological
CIPs	carbonyl iron particles
LDPE	low-density polyethene
